# Activation of melanocortin receptor 4 with RO27-3225 attenuates neuroinflammation through AMPK/JNK/p38 MAPK pathway after intracerebral hemorrhage in mice

**DOI:** 10.1186/s12974-018-1140-6

**Published:** 2018-04-11

**Authors:** Shengpan Chen, Lianhua Zhao, Prativa Sherchan, Yan Ding, Jing Yu, Derek Nowrangi, Jiping Tang, Ying Xia, John H. Zhang

**Affiliations:** 1 Department of Neurosurgery, Affiliated Haikou Hospital, Xiangya School of Medicine, Central South University, Haikou, 570208 China; 2 Department of Physiology and Pharmacology, School of Medicine, Loma Linda University, Loma Linda, CA 92354 USA; 3 Department of Neurosurgery and Anesthesiology, Loma Linda University Medical Center, Loma Linda, CA 92354 USA; 4 Department of Neurology, Tianjin TEDA Hospital, Tianjin, 300457 China

**Keywords:** Intracerebral hemorrhage, Melanocortin receptor 4, RO27-3225, Brain edema, Neuroinflammation

## Abstract

**Background:**

Neuroinflammation plays an important role in the pathogenesis of intracerebral hemorrhage (ICH)-induced secondary brain injury. Activation of melanocortin receptor 4 (MC4R) has been shown to elicit anti-inflammatory effects in many diseases. The objective of this study was to explore the role of MC4R activation on neuroinflammation in a mouse ICH model and to investigate the contribution of adenosine monophosphate-activated protein kinase (AMPK)/c-Jun N-terminal kinase (JNK)/p38 mitogen-activated protein kinase (p38 MAPK) pathway in MC4R-mediated protection.

**Methods:**

Adult male CD1 mice (*n* = 189) were subjected to intrastriatal injection of bacterial collagenase or sham surgery. The selective MC4R agonist RO27-3225 was administered by intraperitoneal injection at 1 h after collagenase injection. The specific MC4R antagonist HS024 and selective AMPK inhibitor dorsomorphin were administered prior to RO27-3225 treatment to elucidate potential mechanism. Short- and long-term neurobehavioral assessments, brain water content, immunofluorescence staining, and western blot were performed.

**Results:**

The expression of MC4R and p-AMPK increased after ICH with a peak at 24 h. MC4R was expressed by microglia, neurons, and astrocytes. Activation of MC4R with RO27-3225 improved the neurobehavioral functions, decreased brain edema, and suppressed microglia/macrophage activation and neutrophil infiltration after ICH. RO27-3225 administration increased the expression of MC4R and p-AMPK while decreasing p-JNK, p-p38 MAPK, TNF-α, and IL-1β expression, which was reversed with inhibition of MC4R and AMPK.

**Conclusions:**

Our study demonstrated that activation of MC4R with RO27-3225 attenuated neuroinflammation through AMPK-dependent inhibition of JNK and p38 MAPK signaling pathway, thereby reducing brain edema and improving neurobehavioral functions after experimental ICH in mice. Therefore, the activation of MC4R with RO27-3225 may be a potential therapeutic approach for ICH management.

**Electronic supplementary material:**

The online version of this article (10.1186/s12974-018-1140-6) contains supplementary material, which is available to authorized users.

## Background

Spontaneous intracerebral hemorrhage (ICH) is a common fatal stroke subtype that accounts for 10 to 15% of all stroke cases and associated with high mortality and morbidity [1]. The hematoma formation and its expansion within the brain parenchyma is regarded as the primary brain injury to damage the brain tissue. Red blood cell lysis, blood products, and thrombin can then initiate inflammatory cell activation which contributes to neuroinflammation, a major contributor to secondary brain injury after ICH that results in brain edema, disruption of the blood-brain barrier, and cell death [2, 3]. Moreover, numerous studies have demonstrated the critical role of inflammation in ICH-induced secondary brain injury, including microglia/macrophage activation and neutrophil infiltration [4–6]. Therefore, anti-inflammatory strategies may have potential therapeutic effects against ICH and improve neurological functions after ICH [7].

Melanocortin receptor 4 (MC4R) is a G protein-coupled receptor with seven transmembrane domains and an intronless gene that encodes a protein of 332 amino acids with four potential glycosylation sites and two potential palmitoylation sites [8, 9]. Among the melanocortin receptors, MC4R is predominantly expressed in the central nervous system (CNS) including the thalamus, hypothalamus, cortex, hippocampus, and brainstem, although it is also detected in peripheral tissues [10, 11]. Moreover, several studies have described that MC4R could be expressed by neurons, microglia, and astrocytes [12, 13]. The binding of MC4R to its endogenous ligand, α-melanocyte-stimulating hormone (α-MSH), has demonstrated the protective, anti-inflammatory, and anti-apoptotic effects in experimental renal ischemia/reperfusion, cerebral ischemia, and traumatic brain injury [14–16]. In recent years, some low molecular weight non-peptide compounds, selective MC4R agonists, appear to be suited for the treatment of immune-mediated inflammatory diseases, without having the side effects of corticosteroids [17, 18]. However, the potential role of MC4R activation against neuroinflammation after ICH-induced brain injury still has not been studied.

c-Jun N-terminal kinase (JNK) and p38 mitogen-activated protein kinase (p38 MAPK) that belong to the MAPK family have already been widely investigated for their roles in response to various stress stimuli [19]. Several studies have demonstrated that JNK and p38 MAPK signaling pathways were activated after ICH, leading to the upregulation of proinflammatory mediators including TNF-α, IL-6, and IL-1β [20, 21]. A previous study indicated that activation of MC4R could significantly decrease the expression levels of JNK and p38 MAPK [18]. Adenosine monophosphate-activated protein kinase (AMPK) plays an important role in energy homeostasis and regulation of inflammatory responses [22]. In addition, MC4R activation was able to attenuate oxidative stress and mitochondrial dysfunction via increasing the expression of AMPK [23]. Recent studies reported that activation of AMPK was shown to rapidly inhibit proinflammatory JNK and p38 MAPK [24, 25].

Therefore, the aim of the present study was to investigate whether the selective MC4R agonist, RO27-3225, could improve neurological outcomes and attenuate neuroinflammation through AMPK/JNK/p38 MAPK pathway in an experimental ICH model.

## Methods

### Animals

A total of 189 male CD1 mice (8-week-old, weight 30–40 g; Charles River, Wilmington, MA) were housed in a temperature and humidity controlled room for a minimum of 3 days before ICH induction with a 12-h light/dark cycle and had access to food and water ad libitum. All the experimental protocols and procedures for this study were approved by the Institutional Animal Care and Use Committee at Loma Linda University and were in compliance with the National Institutes of Health’s Guide for the Care and Use of Laboratory Animals. The manuscript adheres to the ARRIVE (Animal Research: Reporting of In Vivo Experiments) guidelines for reporting animal experiments.

### Experimental protocol

In the present study, all mice were randomly assigned to the following four separate experiments which are shown in the timeline of experimental design (Additional file 1: Figure S1). The experimental groups and number of animals used in experiments 1 to 4 are listed in a table (Additional file 2: Table S1).

#### Experiment 1

To determine the time course of endogenous MC4R and AMPK expressions after ICH, 36 mice were randomly divided into six groups: sham (*n* = 6), 3 h after ICH (*n* = 6), 6 h after ICH (*n* = 6), 12 h after ICH (*n* = 6), 24 h after ICH (*n* = 6), and 72 h after ICH (*n* = 6). Western blot analysis was used to detect their expression in the ipsilateral/right hemisphere of each group. The cellular localization of MC4R was evaluated using double-labeling immunofluorescence labeling to co-localize MC4R with ionized calcium-binding adaptor molecule 1 (Iba-1), neuronal specific nuclear protein (NeuN), and glial fibrillary acidic protein (GFAP) at 24 h after ICH (*n* = 2).

#### Experiment 2

To evaluate the anti-inflammatory effects of intraperitoneal administration of MC4R-selective agonist RO27-3225 at 1 h after ICH, brain water content and neurobehavioral tests were performed at 24 and 72 h after ICH. For effects of the drug at 24 h after ICH, 30 mice were assigned into five groups: sham (*n* = 6), ICH + vehicle (*n* = 6), ICH + RO27-3225 60 μg/kg (*n* = 6), ICH + RO27-3225 180 μg/kg (*n* = 6), and ICH + RO27-3225 540 μg/kg (*n* = 6). Based on brain water content and neurobehavioral tests, 180 μg/kg of RO27-3225 treatment group was the best dosage. For the effects of the drug at 72 h after ICH, 18 mice were randomly divided into three groups: sham (*n* = 6), ICH + vehicle (*n* = 6), and ICH + RO27-3225 180 μg/kg (*n* = 6).

To explore the effects of RO27-3225 administration on microglia/macrophage activation and neutrophil infiltration at 24 h after ICH, 18 mice were randomly divided into three groups for immunofluorescence staining: sham (*n* = 6), ICH + vehicle (*n* = 6), and ICH + RO27-3225 180 μg/kg (*n* = 6). Immunofluorescence staining of Iba-1 and myeloperoxidase (MPO) was performed, and quantitative analysis of Iba-1 and MPO-positive cells were counted in the perihematomal area at 24 h after ICH. Eighteen mice were randomly divided into three groups for western blot analysis: sham (*n* = 6), ICH + vehicle (*n* = 6), and ICH + RO27-3225 180 μg/kg (*n* = 6). Western blot analysis was performed to quantify the expression of Iba-1 and MPO among the three groups at 24 h after ICH.

#### Experiment 3

To assess the effects of RO27-3225 on long-term neurobehavioral functions after ICH, 24 mice were randomly divided into three groups: sham (*n* = 8), ICH + vehicle (*n* = 8), and ICH + RO27-3225 (180 μg/kg) (*n* = 8). The foot fault test and the Rotarod test were performed on days 7, 14, and 21 after ICH while Morris water maze was conducted on days 21–25 after ICH.

#### Experiment 4

To explore the underlying mechanisms of RO27-3225-mediated anti-inflammatory effects after ICH, the selective AMPK inhibitor dorsomorphin was administered by intracerebroventricular injection 30 min before ICH induction, and the specific MC4R antagonist HS024 was administered by intraperitoneal injection 20 min after ICH induction, then followed with RO27-3225 (180 μg/kg) treatment 1 h after ICH. Mice were randomly divided into seven groups: sham (*n* = 6), ICH + vehicle (*n* = 6), ICH + RO27-3225 (*n* = 6), ICH + RO27-3225 + HS024 (*n* = 6), ICH + RO27-3225 + saline (*n* = 6), ICH + RO27-3225 + dorsomorphin (*n* = 6), and ICH + RO27-3225 + DMSO (*n* = 6). The samples for the sham (*n* = 6), ICH + vehicle (*n* = 6), and ICH + RO27-3225 (*n* = 6) groups were shared from experiment 2, and an additional of 24 mice were added. Neurobehavioral tests and western blot analysis were performed at 24 h after ICH.

### Drug administration

RO27-3225 (Sigma-Aldrich, St. Louis, MO) as a selective agonist of MC4R was dissolved in saline and tested at three different doses (60 μg/kg, 180 μg/kg, and 540 μg/kg), which were administered intraperitoneally at 1 h after ICH [17, 18]. HS024 (130 μg/kg; Tocris, Minneapolis, MN) as a specific antagonist of MC4R was dissolved in saline and then administered intraperitoneally 20 min after ICH [26]. The mice of ICH + vehicle group and ICH + RO27-3225 + saline group received an equal volume of saline. The selective AMPK inhibitor, dorsomorphin (5 μg/mouse; Sigma-Aldrich, St. Louis, MO), was dissolved in 5% dimethyl sulfoxide (DMSO) and then injected intracerebroventricularly 30 min before ICH induction [27, 28]. The control group was given the same volume of 5% DMSO by intracerebroventricular injection.

### ICH model

Intracerebral hemorrhage model was induced in mice by intrastriatal injection of bacterial collagenase, as previously reported [29]. Briefly, mice were anesthetized with a mixture of ketamine (100 mg/kg) and xylazine (10 mg/kg) (2:1, intraperitoneal injection) and positioned prone in a stereotaxic head frame (Kopf Instruments, Tujunga, CA), and an artificial tears ointment (Rugby, Livonia, MI) was used for keeping the eyes moist during surgery. A 1-mm cranial burr hole was drilled, and a 26-gauge needle on a 10-μl Hamilton syringe was inserted stereotactically into the right basal ganglia (coordinates 0.2 mm posterior, 2.2 lateral to the bregma, and 3.5 mm below the dura). Bacterial collagenase type VII-S (0.075 units) (Sigma-Aldrich, St. Louis, MO) dissolved in 0.5 μl sterile phosphate-buffered saline (PBS) was infused into the brain at a rate of 0.167 μl/min with an infusion pump (Stoelting, Harvard Apparatus, Holliston, MA). The needle was left in place for an additional 5 min after the injection to prevent possible leakage of the collagenase solution and withdrawn slowly at a rate of 1 mm/min. The cranial burr hole was sealed with bone wax, the scalp was sutured, and 0.4 ml of normal saline was injected subcutaneously to avoid postsurgical dehydration. Mice were allowed to recover fully under close observation. The sham operation was performed following the same procedure but with needle insertion only.

### Intracerebroventricular injection

Intracerebroventricular administration was performed as previously described [30]. Briefly, the 26-gauge needle of a 10-μl Hamilton syringe was inserted into the left lateral ventricle through a cranial burr hole at the following coordinates relative to bregma: 0.3 mm posterior, 1.0 lateral, and 2.3 mm deep. A microinfusion pump was used for intracerebroventricular administration at a rate of 0.667 μl/min. The needle was left in place for an additional 8 min after the end of infusion and then removed over 3 min. The burr hole was sealed with bone wax.

### Neurobehavioral function assessment

Short-term neurobehavioral functions were assessed with modified Garcia score, forelimb placement test, and corner turn test by an independent researcher blinded to the experimental groups at 24 and 72 h after ICH, as previously described [31, 32]. The Garcia neuroscore consisted of seven individual tests that evaluated spontaneous activity, axial sensation, vibrissae proprioception, symmetry of limb movement, lateral turning, forelimb walking, and climbing. Each subtest was given a score ranging from 0 to 3, with a composite maximum score of 21 (no neurological deficits). The forelimb placement test was used to assess the animals’ responsiveness to vibrissae stimulation, and results were expressed as a percentage of the number of successful left forepaw placements out of 10 stimulations, normalized to the mean of sham performance. For the corner turn test, animals were allowed to advance into a 30° corner and exit by turning either to the left or right. Choice of turning was recorded for a total of 10 trials, and a score was calculated as number of left turns/all trials ×  100.

Long-term neurobehavioral assessments were performed with foot fault test and Rotarod test to assess sensorimotor coordination and balance in the first, second, and third week after ICH and with Morris water maze to evaluate spatial learning and memory abilities on days 21 to 25 after ICH, as previously reported [33]. Briefly, for foot fault tests, mice were allowed to move along a horizontal wire grid (20 cm × 100 cm) for 2 min. The number of left forelimb missteps was recorded. Rotarod (Columbus Instruments, Columbus, OH) consisted of a 7-cm diameter of rotating horizontal cylinder that was divided into 9.5 cm wide lanes. Mice had to keeping walking forward after being placed on the cylinder. The cylinder started at 5 revolutions per minute (RPM) and was accelerated by 2 RPM every 5 s. The falling latency was recorded by a photobeam circuit. For Morris water maze, mice were required to find the submerged platform, which was recorded by an overhead camera linked to a computer tracking system (Noldus Ethovision, Tacoma, WA). Their swim path, escape latency, and swim distance were recorded individually. The probe trial was performed on the last day of testing in which the platform was removed and the duration of time spent in the probe quadrant was recorded.

### Brain water content measurement

Brain edema was evaluated by measuring brain water content using wet/dry method as previously described [34]. Briefly, mice were decapitated under deep anesthesia at 24 and 72 h after ICH, and the brains were immediately removed and divided into five parts, namely, the ipsilateral and contralateral cortices, ipsilateral and contralateral basal ganglia, and cerebellum (which served as an internal control). Each part was immediately weighed on an electric analytic balance (APX-60, Denver Instrument) to obtain the wet weight and then dried at 100 °C for 24 h to obtain the dry weight. Brain water content was calculated using the following formula: brain water content (%) = [(wet weight − dry weight)/wet weight] × 100%.

### Western blot analysis

Western blot was performed as previously described [35]. Mice were transcardially perfused with ice-cold PBS under deep anesthesia, and the brains were removed and separated into two hemispheres at 24 h after ICH or sham surgeries. The ipsilateral/right brain hemispheres were homogenized in RIPA lysis buffer (Santa Cruz Biotechnology, Santa Cruz, CA) and centrifuged at 4 °C for 30 min at 14,000*g*. The supernatant was collected, and the protein concentration was determined using a detergent compatible assay (DC protein assay, Bio-Rad laboratories, CA). Equal amounts of protein were loaded on an SDS-PAGE gel and run using electrophoresis and then transferred to a nitrocellulose membrane. The membrane was blocked and incubated at 4 °C overnight with the following primary antibodies: rabbit anti-MC4R (1:500, Abcam, Cambridge, MA), rabbit anti-AMPK (1:1000, Cell Signaling, Danvers, MA), rabbit anti-phosphorylated AMPK (p-AMPK, Thr 172, 1:1000, Cell Signaling), rabbit anti-JNK (1:1000, Abcam), rabbit anti-phosphorylated JNK (p-JNK, 1:1000, Abcam), mouse anti-p38 (1:300, Santa Cruz Biotechnology, Santa Cruz, CA), mouse anti-phosphorylated p38 (p-p38, 1:300, Santa Cruz), goat anti-Iba-1 (1:1000, Abcam), rabbit anti-MPO (1:500, Abcam), rabbit anti-TNF-α (1:1000, Abcam), rabbit anti-IL-1β (1:1000, Abcam), and goat anti-actin (1:5000, Santa Cruz). Appropriate secondary antibodies (1:3000, Santa Cruz; 1:5000, Abcam) were selected to incubate with the membrane for 2 h at room temperature. The bands were probed with an ECL Plus chemiluminescence regent Kit (Amersham Biosciences, Arlington Heights, PA) and visualized with the image system (Bio-Rad, Versa Doc, model 4000). Relative density of the protein immunoblot images were analyzed by ImageJ software (ImageJ 1.4, NIH, USA).

### Immunofluorescence staining

Mice were perfused under deep anesthesia with ice-cold PBS followed by perfusion with 10% formalin at 24 h after surgeries. The brains were removed and fixed in formalin at 4 °C overnight and dehydrated with 30% sucrose for 3 days. Brain samples were then snap-frozen at − 80 °C and cut into 10-μm-thick coronal sections using a cryostat (CM3050S; Leica Microsystems). Immunofluorescence staining was performed as previously described [36]. Briefly, brain samples were incubated overnight at 4 °C with primary antibodies including goat anti-Iba-1 (1:100, Abcam), goat anti-GFAP (1:100, Abcam), goat anti-NeuN (1:200, Abcam), rabbit anti-MC4R (1:500, Abcam), and rabbit anti-MPO (1:500, Abcam). The sections were then incubated with corresponding secondary antibodies (1:200, Jackson Immunoresearch, West Grove, PA) at room temperature for 2 h and followed by visualization using a fluorescence microscope (Leica Microsystems, Germany).

### Statistical analysis

All data were expressed as the mean and standard deviation (mean ± SD). Statistical analysis was performed with Graph Pad Prism (Graph Pad Software, San Diego, CA). One-way analysis of variance (ANOVA) is followed by multiple comparisons between groups using Tukey’s post hoc test. Two-way repeated measures ANOVA was used to analyze the long-term neurobehavioral functions over time. Statistical significance was set at *p* < 0.05.

## Results

### Animal mortality and exclusion

None of the sham-operated mice died in this study. The total animal mortality rate of the study was 10.60% (16/151). The mortality rate was not significantly different between the experimental groups. Three mice were excluded from the study because of no hemorrhage (Additional file 2: Table S1).

### Time course and spatial expressions of MC4R and phosphorylated AMPK after ICH

**Fig. 1:**
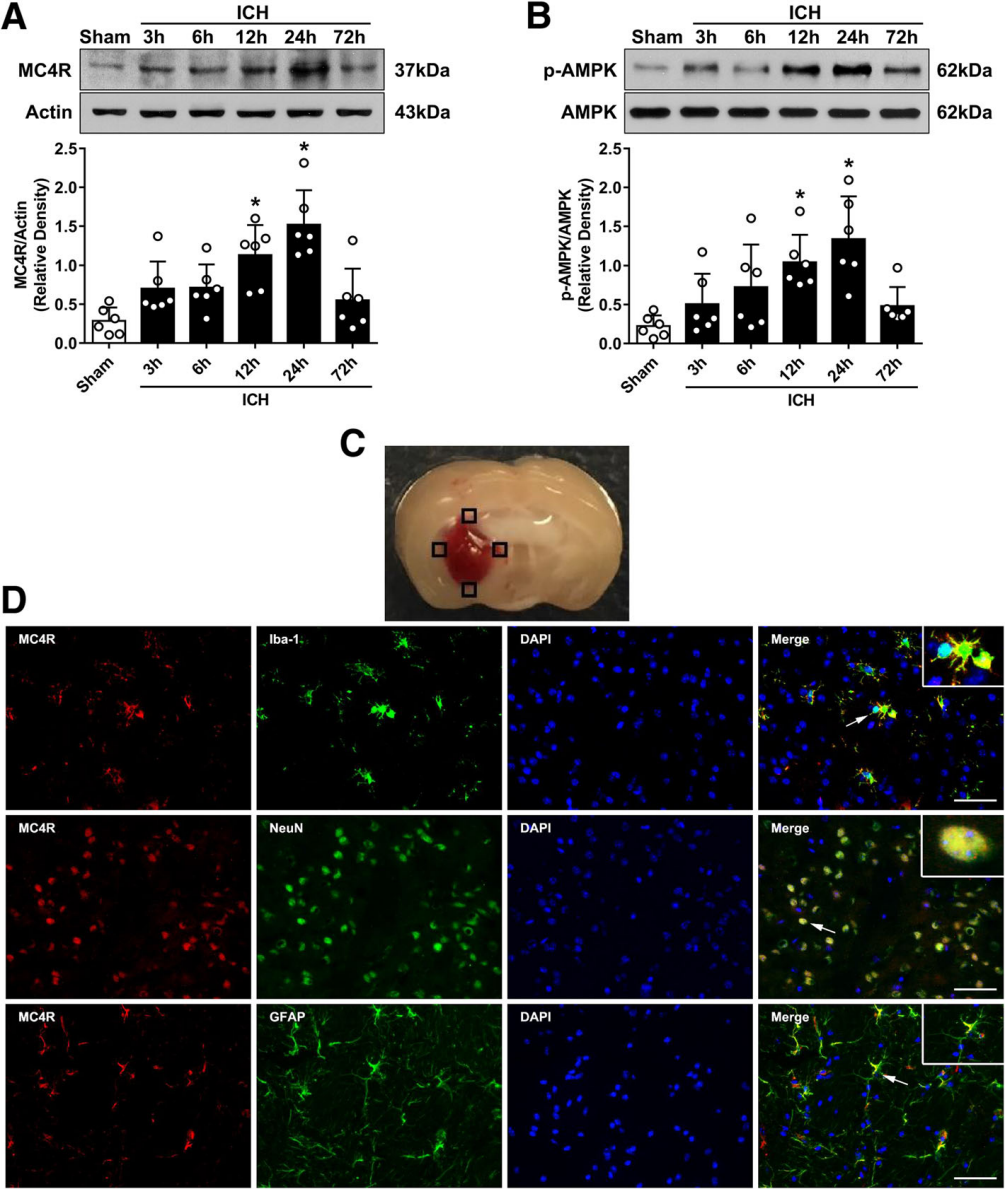
Expression profile of MC4R and p-AMPK after ICH. **a** Representative western blot bands of time course and quantitative analyses of MC4R expression in the ipsilateral hemisphere after ICH. **b** Representative western blot bands of time course and quantitative analyses of phosphorylated AMPK expression in the ipsilateral hemisphere after ICH. *p < 0.05 vs sham. Error bars are represented as mean ± SD. One-way ANOVA, Tukey’s test, *n* = 6 per group. **c** Brain sample with schematic illustration showing the four areas in the perihematomal region (indicated by black squares) from where the images were taken for immunofluorescence staining (pictures are shown in panel **d**). **d** Representative images of co-localization of MC4R (red) with microglia/macrophage (Iba-1, green), neurons (NeuN, green), and astrocytes (GFAP, green) in the perihematomal area at 24 h after ICH. Nuclei were stained with DAPI (blue). Scale bar = 50 μm, *n* = 2

Western blot was performed to assess the protein expression of MC4R and phosphorylated AMPK at 0, 3, 6, 12, 24, and 72 h in the ipsilateral/right cerebral hemispheres after ICH. The results showed that the expression of MC4R and phosphorylated AMPK increased as early as 3 h, reached the peak at 24 h, and decreased at 72 h after ICH (*p* < 0.05, Fig. 1a, b). Double immunofluorescence staining was performed to detect the localization of MC4R on microglia/macrophage (Iba-1), neurons (NeuN), and astrocytes (GFAP) at 24 h after ICH. These images showed that MC4R was expressed on these three cell types at 24 h after ICH (Fig. 1c).

### RO27-3225 treatment improved neurobehavioral outcomes and reduced brain edema at 24 and 72 h after ICH

**Fig. 2:**
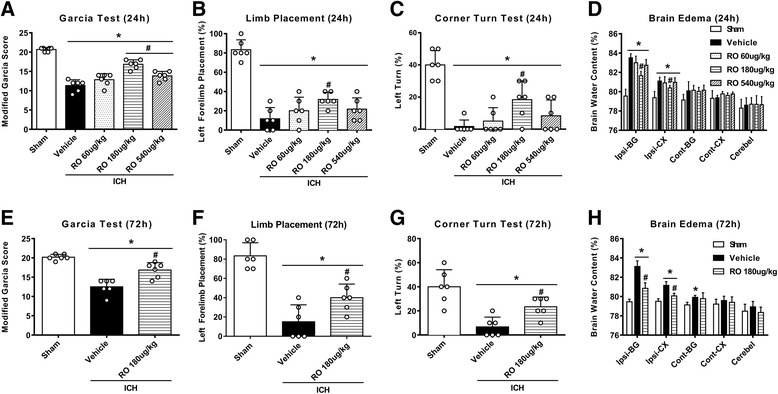
The effects of RO27-3225 on neurobehavioral outcomes and brain water content after ICH. **a** Modified Garcia test, **b** forelimb placement test, **c** corner turn test, and **d** brain water content at 24 h after ICH. **e** Modified Garcia test, **f** forelimb placement test, **g** corner turn test, and **h** brain water content at 72 h after ICH. *p < 0.05 vs. sham, ^**#**^*p* < 0.05 vs. ICH + vehicle. Error bars are represented as mean ± SD. One-way ANOVA, Tukey’s test, *n* = 6 per group. RO RO27-3225, Ipsi-BG ipsilateral basal ganglia, Ipsi-CX ipsilateral cortex, Cont-BG contralateral basal ganglia, Cont-CX contralateral cortex, Cerebel cerebellum

Neurobehavioral outcomes and brain water content were evaluated at 24 and 72 h after ICH. Mice in ICH groups performed significantly worse than the sham in the neurobehavioral tests including the modified Garcia test (*p* < 0.05, Fig. 2a, e), forelimb placement test (*p* < 0.05, Fig. 2b, f), and corner turn test (*p* < 0.05, Fig. 2c, g) at 24 and 72 h after ICH. The administration of RO27-3225 (180 μg/kg) significantly improved performance in all three neurobehavioral tests at 24 and 72 h after ICH (*p* < 0.05, Fig. 2a–c, e–g). Brain water content in the ipsilateral basal ganglia and ipsilateral cortex was significantly increased in the ICH groups compared to sham at 24 and 72 h after ICH (*p* < 0.05, Fig. 2d, h), which was significantly reduced with the administration of RO27-3225 (180 μg/kg) (*p* < 0.05, Fig. 2d, h). Therefore, the medium dosage of RO27-3225 was chosen for 72 h, long-term and mechanistic studies.

### RO27-3225 treatment inhibited microglia/macrophage activation and neutrophil infiltration after ICH

**Fig. 3:**
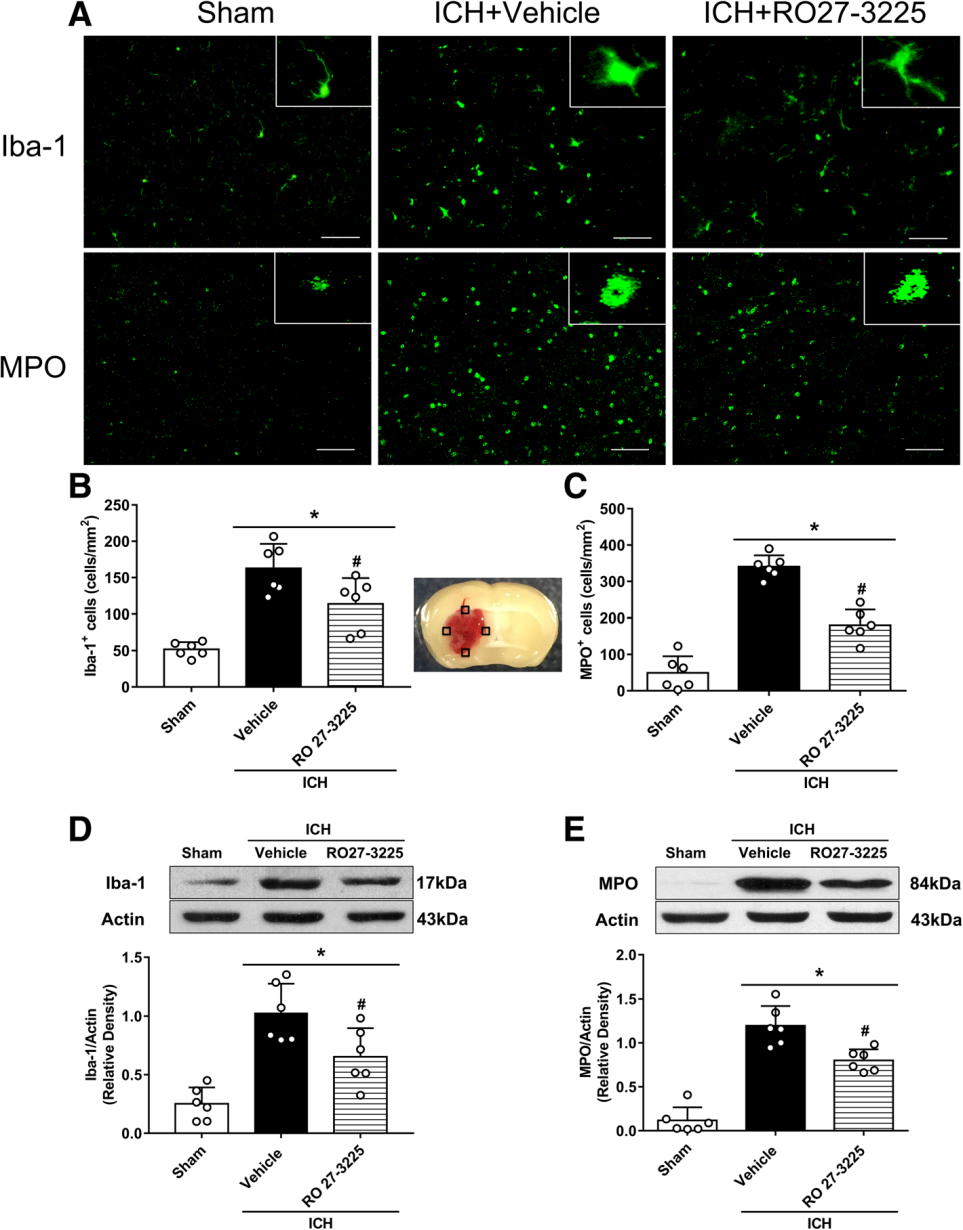
The effects of RO27-3225 on microglia/macrophage activation and neutrophil infiltration after ICH. **a** Representative images of immunofluorescence staining of Iba-1 (green) and MPO (green) in the perihematomal area at 24 h after ICH. Brain sample with schematic illustration shows the four areas (indicated by black squares) used for Iba-1 and MPO-positive cell counting in the perihematomal region. **b**, **c** Quantitative analyses of Iba-1 and MPO-positive cells in the perihematomal area at 24 h after ICH. **d**, **e** Representative western blot bands and quantitative analyses of Iba-1 and MPO protein levels in the ipsilateral hemisphere at 24 h after ICH. *p < 0.05 vs. sham, ^#^*p* < 0.05 vs. ICH + vehicle. Error bars are represented as mean ± SD. One-way ANOVA, Tukey’s test, *n* = 6 per group

Iba-1 and MPO levels in the brain were performed to detect microglia/macrophage activation and neutrophil infiltration by immunofluorescence staining and western blot in sham, ICH + vehicle, and ICH + RO27-3225 groups at 24 h after ICH. Immunofluorescence staining indicated that RO27-3225 treatment significantly decreased the number of Iba-1 and MPO-positive cells in the perihematomal area compared to the ICH + vehicle group at 24 h after ICH (*p* < 0.05, Fig. 3a–c). Consistently, western blot results showed that the expression levels of Iba-1 and MPO in the ipsilateral hemisphere were significantly decreased with RO27-3225 treatment compared to those in the vehicle-treated animals at 24 h after ICH (*p* < 0.05, Fig. 3d, e).

### RO27-3225 treatment improved long-term movement coordination ability, spatial learning, and memory abilities after ICH

**Fig. 4:**
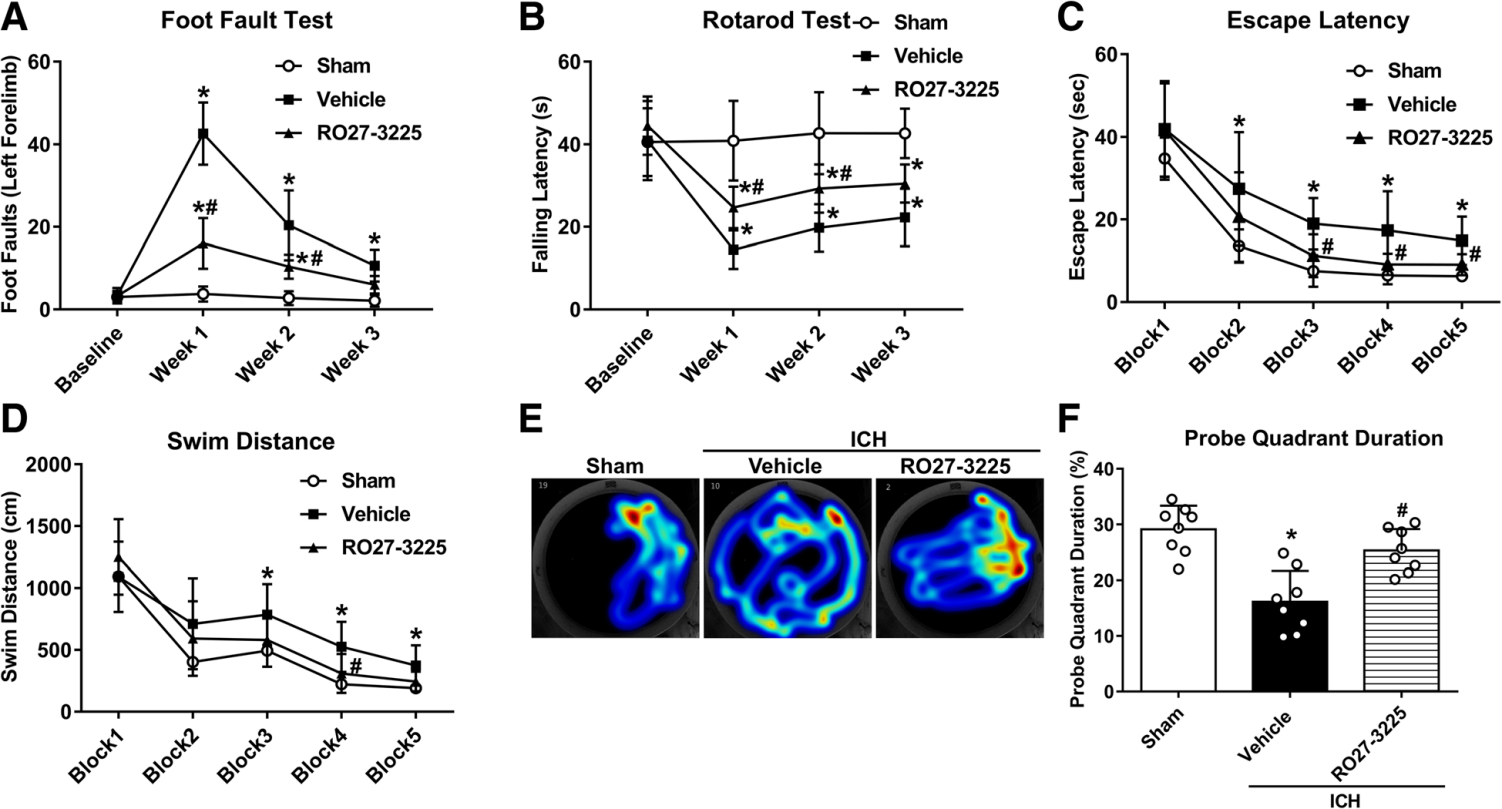
The effects of RO27-3225 on long-term neurobehavioral outcomes after ICH. **a** Foot fault test and **b** Rotarod test in the first, second, and third week after ICH. **c** Escape latency and **d** swim distance of Morris water maze on days 21 to 25 after ICH. **e** Typical traces and **f** probe quadrant duration of Morris water maze on day 25 after ICH. *p < 0.05 vs. sham, ^#^*p* < 0.05 vs. ICH + vehicle. Error bars are represented as mean ± SD. Two-way repeated measures ANOVA, Tukey’s test (**a**–**d**), and one-way ANOVA, Tukey’s test (**f**), *n* = 8 per group

In the foot fault test and Rotarod test, the ICH + vehicle group had significantly more foot faults of the left forelimb and shorter falling latency compared to sham animals in the first, second, and third week after ICH (*p* < 0.05, Fig. 4a, b). However, the ICH + RO27-3225 group had significantly decreased foot faults of the left forelimb and increased falling latency compared to the ICH + vehicle group in the first and second week (*p* < 0.05, Fig. 4a, b).

In the Morris water maze, the escape latency and swim distance for the mice to find the platform were significantly increased in the ICH + vehicle group when compared to the sham group (*p* < 0.05, Fig. 4c, d). However, a significant decrease in escape latency on blocks 3 to 4 and a significantly shorter swim distance on block 4 were observed in the ICH + RO27-3225 group compared to the ICH + vehicle group (*p* < 0.05, Fig. 4c, d). In the probe quadrant trial, the ICH + vehicle group spent less time in the target quadrant when the platform was removed compared to the sham group (*p* < 0.05, Fig. 4e, f), while the time spent in the target quadrant was significantly increased in the ICH + RO27-3225 group compared to the ICH + vehicle group (*p* < 0.05, Fig. 4e, f).

### MC4R antagonist and AMPK inhibitor reversed the protective effects of RO27-3225 on neurobehavioral outcomes after ICH

**Fig. 5:**
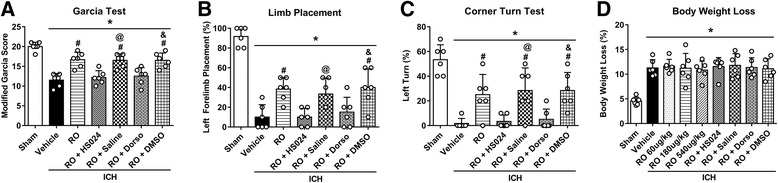
MC4R antagonist and AMPK inhibitor reversed the effects of RO27-3225 on neurobehavioral outcomes after ICH. **a** Modified Garcia test, **b** forelimb placement test, **c** corner turn test, and **d** the effects of RO27-3225 on body weight loss at 24 h after ICH. *p < 0.05 vs. sham, ^#^*p* < 0.05 vs. ICH + vehicle, ^@^*p* < 0.05 vs. ICH + RO27-3225 + HS024, and ^&^*p* < 0.05 vs. ICH + RO27-3225 + DMSO. Error bars are represented as mean ± SD. One-way ANOVA, Tukey’s test, *n* = 6 per group

Treatment with RO27-3225 improved the performance in neurobehavioral tests including the modified Garcia test, forelimb placement test, and corner turn test at 24 h after ICH, which was reversed with the administration of MC4R-selective antagonist HS024 (*p* < 0.05, Fig. 5a, c) as well as with the administration of AMPK-specific inhibitor dorsomorphin (*p* < 0.05, Fig. 5a, c). In addition, administration of RO27-3225, HS024, and dorsomorphin did not result in any significant difference in body weight loss compared to the ICH + vehicle group at 24 h after ICH (*p* < 0.05, Fig. 5d).

### RO27-3225 treatment attenuated neuroinflammation through MC4R/AMPK/JNK/p38 MAPK pathway after ICH

**Fig. 6:**
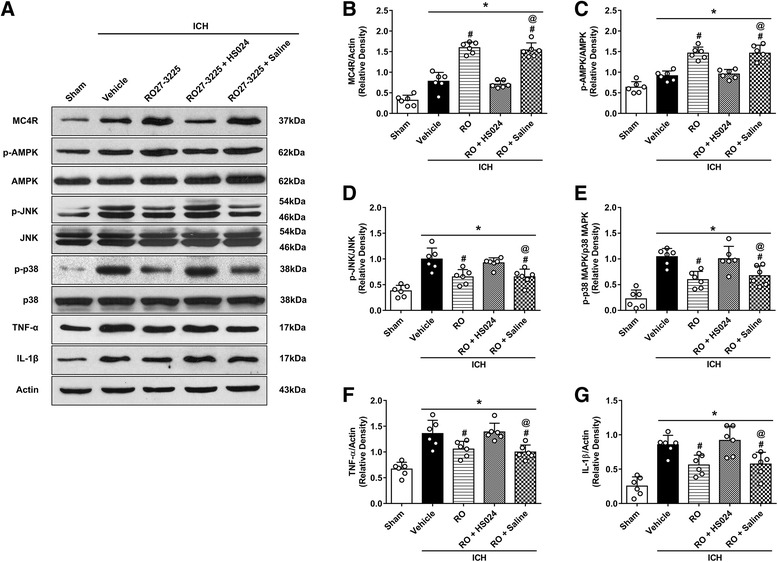
The effects of RO27-3225 and MC4R antagonist HS024 on the expression of MC4R and its downstream signaling proteins. **a** Representative western blot bands. **b**–**g** Quantitative analyses of MC4R, phosphorylated AMPK, phosphorylated JNK, phosphorylated p38 MAPK, TNF-α, and IL-1β in the ipsilateral hemisphere at 24 h after ICH. *p < 0.05 vs. sham, ^#^*p* < 0.05 vs. ICH + vehicle, and ^@^*p* < 0.05 vs. ICH + RO27-3225 + HS024. Error bars are represented as mean ± SD. One-way ANOVA, Tukey’s test, *n* = 6 per group

**Fig. 7:**
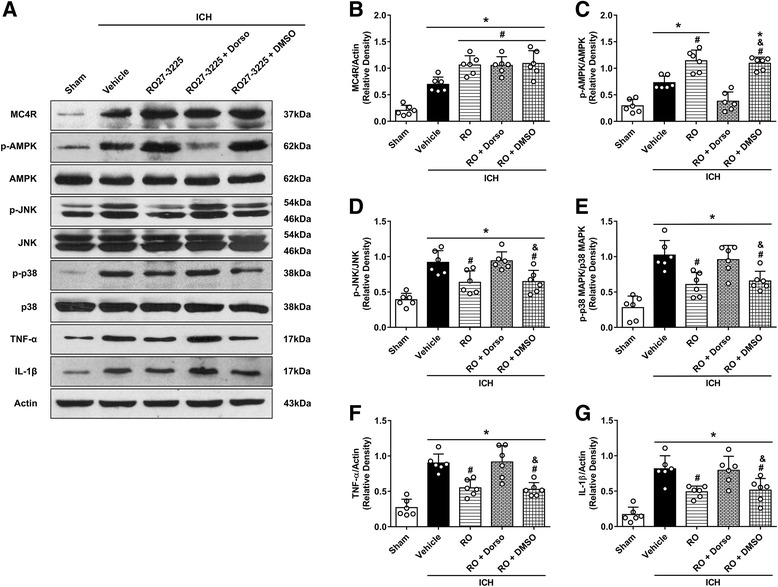
The effects of RO27-3225 and AMPK inhibitor dorsomorphin on the expression of MC4R and its downstream signaling proteins. **a** Representative western blot bands. **b**–**g** Quantitative analyses of MC4R, phosphorylated AMPK, phosphorylated JNK, phosphorylated p38 MAPK, TNF-α, and IL-1β in the ipsilateral hemisphere at 24 h after ICH. *p < 0.05 vs. sham, ^#^*p* < 0.05 vs. ICH + vehicle, and ^&^*p* < 0.05 vs. ICH + RO27-3225 + DMSO. Error bars are represented as mean ± SD. One-way ANOVA, Tukey’s test, *n* = 6 per group

After RO27-3225 treatment, the expression levels of MC4R and phosphorylated AMPK were significantly increased compared to those in the sham group and the ICH + vehicle group at 24 h after ICH (*p* < 0.05, Figs. 6 and 7). Moreover, the expression levels of phosphorylated JNK, phosphorylated p38 MAPK, TNF-α, and IL-1β were significantly decreased in the ICH + RO27-3225 group compared to the ICH + vehicle group at 24 h after ICH (*p* < 0.05, Figs. 6 and 7). However, inhibition of MC4R with HS024 remarkably decreased the expression of MC4R and phosphorylated AMPK, while increased the expression of phosphorylated JNK, phosphorylated p38 MAPK, TNF-α, and IL-1β, compared to the ICH + RO27-3225 + saline group at 24 h after ICH (*p* < 0.05, Fig. 6a–g). Consistently, pretreatment with dorsomorphin significantly decreased the expression of phosphorylated AMPK, while increased the expression of phosphorylated JNK, phosphorylated p38 MAPK, TNF-α, and IL-1β compared to the ICH + RO27-3225 + DMSO group at 24 h after ICH (*p* < 0.05, Fig. 7a–g).

## Discussion

In the present study, we investigated the potential anti-inflammatory effects of activation of MC4R and its underlying mechanism after collagenase-induced ICH in mice. Our results showed that activation of MC4R with its selective agonist RO27-3225 improved the neurobehavioral functions, decreased brain edema, and inhibited microglia/macrophage activation and neutrophil infiltration in perihematomal areas after ICH. In addition, RO27-3225 treatment improved long-term neurobehavioral outcomes after ICH. Mechanistically, administration of RO27-3225 was associated with upregulation of MC4R and phosphorylated AMPK and downregulation of phosphorylated JNK, phosphorylated p38 MAPK, TNF-α, and IL-1β after ICH. However, blockage of MC4R and AMPK reversed the beneficial effects of RO27-3225 on neurobehavioral functions, brain edema, and the inflammatory protein expression. Finally, our findings suggested that activation of MC4R with RO27-3225 might attenuate neuroinflammation after ICH, which was, at least in part, mediated by the AMPK/JNK/p38 MAPK signaling pathway.

Melanocortin neuropeptides, including the adrenocorticotropic hormone (ACTH), α-melanocyte-stimulating hormones (α-MSH), shorter fragments, and synthetic analogs, have significant influences on energy homeostasis, memory formation, cardiovascular regulation, neuroprotection, and inflammation [9, 37]. These peptides act through five melanocortin G protein-coupled receptors MC1R to MC5R, among which MC4R is the predominant melanocortin receptor subtype in the CNS [10]. It has been reported that MC4R was expressed on neurons, microglia, and astrocytes in previous studies [12, 13]. Consistently, our double immunofluorescence staining results showed that MC4R was positively expressed on neurons, microglia, and astrocytes following ICH. Interestingly, we also observed that the expression level of MC4R was upregulated in the right hemisphere in a time-dependent manner after ICH. A previous study demonstrated that the mRNA level of MC4R was increased in the contralateral striatum after hypoxia-ischemic brain injury in rats [38]. Likewise, the mRNA and protein expression of MC4R in the rat liver cells were dramatically increased during acute phase response and liver regeneration [39, 40]. Taken together, these results indicate that MC4R may be increased as a response to deleterious stimuli to counteract cytokine production in the acute phase after ICH.

Numerous studies have indicated that activation of MC4R exerts anti-inflammatory effects by inhibiting the production of proinflammatory cytokines in various diseases, including circulatory shock, myocardial ischemia, and ischemic stroke [18, 41–43]. In recent years, apart from α-MSH and its synthetic analogues, the selective MC4R small molecule agonist RO27-3225 was used to activate MC4R. A previous study reported that RO27-3225 attenuated the inflammatory and apoptotic responses and improved neuronal functionality after cerebral ischemia in gerbils [18]. Moreover, the anti-inflammatory and protective effects of RO27-3225 have been shown in brain injuries induced by intraabdominal hypertension, pancreatitis severity, and hemorrhage shock [17, 44–46]. Up to date, our study is the first to administrate RO27-3225 to determine whether it has protective effects in experimental ICH.

It is well known that brain edema is a pathological phenomenon associated with hematoma enlargement, which results in poor neurological outcomes of ICH [5]. We found that RO27-3225 treatment ameliorated the neurobehavioral impairment and reduced brain edema at 24 and 72 h after ICH. Furthermore, the sensorimotor (foot fault test and Rotarod test) and cognitive (Morris water maze) assessments were also performed in our long-term study. Our results showed that the administration of RO27-3225 has the potential to improve long-term movement coordination ability, spatial learning, and memory abilities after collagenase-induced ICH. In addition, mounting evidence suggests that microglial activation and neutrophil infiltration after ICH exacerbated the release of proinflammatory mediators, such as TNF-α and IL-1β, reactive oxygen species (ROS), nitric oxide, and other potentially toxic factors, leading to neuroinflammation and ICH-induced secondary brain injuries [5, 6, 47]. On the contrary, several studies have reported that activation of MC4R obviously inhibited the overexpression of TNF-α, IL-6, and IL-1β in cerebral ischemia, Alzheimer’s disease, and subarachnoid hemorrhage [8, 18]. Consistent with previous findings, our data showed that RO27-3225 significantly inhibited microglia/macrophage activation and neutrophil infiltration and downregulated the expression of Iba-1, MPO, TNF-α, and IL-1β after ICH. In general, our present study indicated that activation of MC4R with RO27-3225 exerted an anti-inflammatory effect in ICH mice.

Next, we evaluated the possible mechanism by which MC4R activation reduces neuroinflammation after ICH. Recent studies have demonstrated the role of AMPK as a novel signaling molecule modulating inflammatory response and oxidative stress in various cell types and animal models [48]. AMPK is generally activated under conditions, such as glucose deprivation, exercise, oxidative stress, CA^2+^ overload, and ischemia [22]. Activation of AMPK was shown to suppress the expression of JNK and p38 MAPK [24, 25]. Moreover, a recent study showed that MC4R activation attenuated oxidative stress and mitochondrial dysfunction via increasing AMPK [23]. Additionally, the activation of MC4R could also significantly decrease the expression levels of JNK and p38 MAPK [18] and further inhibited the proinflammatory mediators TNF-α and IL-1β, as well as the proapoptotic marker Bax [18, 49]. Consistently, our western blot results demonstrated that RO27-3225 treatment upregulated the expression of MC4R and phosphorylated AMPK, while downregulated the expression of phosphorylated JNK, phosphorylated p38 MAPK, TNF-α, and IL-1β after ICH. To further validate this pathway, HS024 and dorsomorphin were employed to investigate the neurobehavioral functions and the expression of downstream signaling proteins. Our results showed that pretreatment with HS024 and dorsomorphin abolished the protective effects of RO27-3225 on neurobehavioral functions. Furthermore, blockage of those two proteins significantly upregulated the expressions of phosphorylated JNK, phosphorylated p38 MAPK, and proinflammatory cytokines TNF-α and IL-1β. Thus, these findings suggested that activation of MC4R with RO27-3225 may alleviate the neuroinflammation through inhibition of JNK and p38 MAPK dependent on AMPK activation after collagenase-induced ICH.

There are still some limitations in this study. First, since we only focused on the anti-inflammatory effects of activation of MC4R after ICH, we cannot exclude the possibility that activation of MC4R may have also exerted other protective effects, such as anti-apoptosis, preservation of blood-brain barrier integrity, and synaptic plasticity [18, 50]. Thus, further studies are needed to investigate those effects of activation of MC4R after ICH and its underlying signaling mechanisms. Second, previous studies have reported that MC4R also activates the Gs/cAMP/PKA pathway to alleviate the inflammation [8]. We cannot rule out the possibility that other pathways may also contribute to downregulating inflammatory mediators with the activation of MC4R. Besides, the reason why activation of MC4R regulates the expression of AMPK after ICH is still unclear. Further studies are needed to clarify this mechanism. Last but not least, MC4R is involved in the regulation of food intake and body weight in a dose-dependent manner [51]. Although the single administration of RO27-3225 did not significantly affect the body weight at 24 h after ICH in our study, whether long-term administration of this drug can change the body weight should be closely observed in the future.

## Conclusions

Our study demonstrated that activation of MC4R with RO27-3225 can improve neurobehavioral functions and attenuate brain edema and neuroinflammation through the AMPK/JNK/p38 MAPK signaling pathway in an experimental ICH model. Therefore, activation of MC4R with RO27-3225 may be a promising therapeutic strategy in ICH management.

## Additional files

Additional file 1: **Figure S1.** Experimental design and animal groups. ICH, intracerebral hemorrhage; WB, western blot; IHC, immunohistochemistry; DMSO, dimethyl sulfoxide. The asterisk indicates samples shared with experiment 2. (TIFF 2095 kb)

Additional file 2: **Table S1.** Summary of experimental groups and mortality rate in the study. (DOCX 15 kb)

## Abbreviations

ACTH: Adrenocorticotrophic hormone
AMPK: Adenosine monophosphate-activated protein kinase
ARRIVE: Animal Research: Reporting of In Vivo Experiments
Cerebel: Cerebellum
CNS: Central nervous system
Cont-BG: Contralateral basal ganglia
Cont-CX: Contralateral cortex
GFAP: Glial fibrillary acidic protein
Iba-1: Ionized calcium-binding adaptor molecule
ICH: Intracerebral hemorrhage
Ipsi-BG: Ipsilateral basal ganglia
Ipsi-CX: Ipsilateral cortex
JNK: c-Jun N-terminal kinase
MC4R: Melanocortin receptor 4
MPO: Myeloperoxidase
NeuN: Neuronal specific nuclear protein
p38 MAPK: p38 mitogen-activated protein kinase
PBS: Phosphate-buffered saline
RO: RO27-3225
ROS: Reactive oxygen species
RPM: Revolutions per minute
α-MSH: α-Melanocyte-stimulating hormone
