# Supplementary figures and images for: Activation of melanocortin receptor 4 with RO27-3225 attenuates neuroinflammation through AMPK/JNK/p38 MAPK pathway after intracerebral hemorrhage in mice
Source: J Neuroinflammation. 2018 Apr 11;15:106. doi: 10.1186/s12974-018-1140-6 (PMC5896146; doi:10.1186/s12974-018-1140-6)

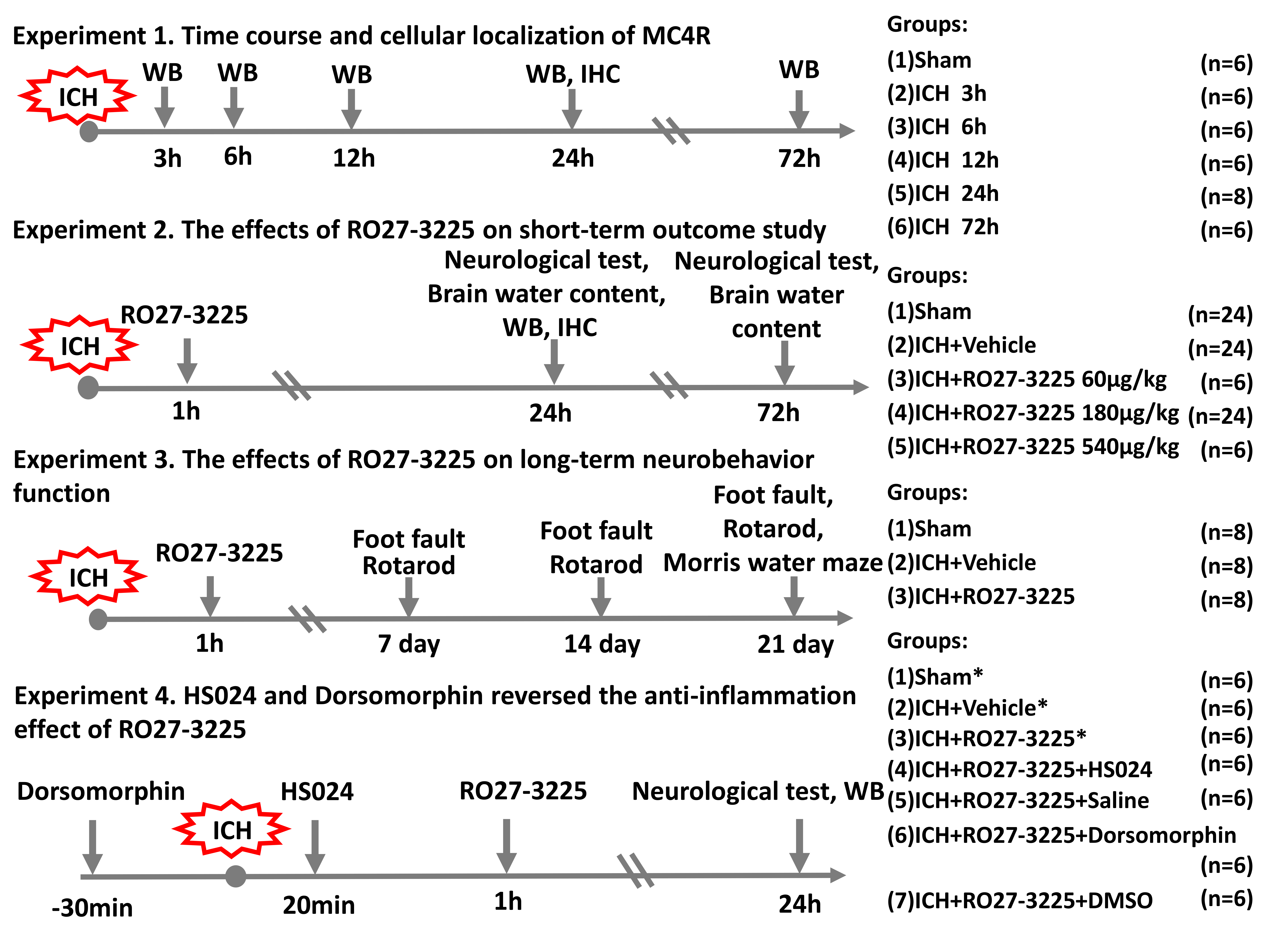

Supplement: Supplementary file 1 — Figure S1. Experimental design and animal groups. ICH, intracerebral hemorrhage; WB, western blot; IHC, immunohistochemistry; DMSO, dimethyl sulfoxide. The asterisk indicates samples shared with experiment 2. (TIFF 2095 kb) [file 12974_2018_1140_MOESM1_ESM.tif]
