# Supplementary material for: Activation of melanocortin receptor 4 with RO27-3225 attenuates neuroinflammation through AMPK/JNK/p38 MAPK pathway after intracerebral hemorrhage in mice
Source: J Neuroinflammation. 2018 Apr 11;15:106. doi: 10.1186/s12974-018-1140-6 (PMC5896146; doi:10.1186/s12974-018-1140-6)
Supplement: Supplementary file 2 — Table S1. Summary of experimental groups and mortality rate in the study. (DOCX 15 kb) [file 12974_2018_1140_MOESM2_ESM.docx]

| **Table S1.** Summary of experimental groups and mortality rate in the study. | | | | | | |
| --- | --- | --- | --- | --- | --- | --- |
|  |  |  |  |  |  |  |
| **Experimental Groups** | **Neurological test** | **IHC** | **WB** | **Exclusion** | **Mortality** | **Subtotal** |
|  | **Brain water content** |  |  |  | **(%)** |  |
| **Experimental 1** |  |  |  |  |  |  |
| Sham |  |  | 6 | 0 | 0 | 6 |
| ICH (3h, 6h, 12h, 24h, 72h) |  | 2 | 30 | 1 | 4(10.81%) | 37 |
| **Experimental 2** |  |  |  |  |  |  |
| Sham | 12 | 6 | 6 | 0 | 0 | 24 |
| ICH + Vehicle | 12 | 6 | 6 | 1 | 3(10.71%) | 28 |
| ICH + RO27-3225 60μg/kg | 6 |  |  | 0 | 1(14.29%) | 7 |
| ICH + RO27-3225 180μg/kg | 12 | 6 | 6 | 0 | 2(7.69%) | 26 |
| ICH + RO27-3225 540μg/kg | 6 |  |  | 0 | 1(14.29%) | 7 |
| **Experimental 3** |  |  |  |  |  |  |
| Sham | 8 |  |  | 0 | 0 | 8 |
| ICH + Vehicle | 8 |  |  | 0 | 0 | 8 |
| ICH + RO27-3225 | 8 |  |  | 1 | 1(10%) | 10 |
| **Experimental 4** |  |  |  |  |  |  |
| ICH + RO27-3225 + HS024 |  |  | 6 | 0 | 2(25%) | 8 |
| ICH + RO27-3225 + Saline |  |  | 6 | 0 | 0 | 6 |
| ICH + RO27-3225 + Dorso |  |  | 6 | 0 | 1(14.29%) | 7 |
| ICH + RO27-3225 + DMSO |  |  | 6 | 0 | 1(14.29%) | 7 |
| **Total** | 72 | 20 | 78 | 3 | 16(10.60%) | 189 |
| ICH, intracerebral hemorrhage; WB, western blot; IHC, immunohistochemistry; Dorso, Dorsomorphin; DMSO, dimethyl sulfoxide | | | | | | |
